# The Pivotal Role of Advanced Echocardiography in Transcatheter Closure of Challenging Secundum Atrial Septal Defect Anatomies: An Expert-Based Review

**DOI:** 10.3390/jcdd13060261

**Published:** 2026-06-11

**Authors:** Bushra Shahida Rana, Brian Clapp, Iqbal Saeed Malik

**Affiliations:** 1 St Thomas’ and Royal Brompton Hospital, Guy’s and St Thomas Hospitals NHS Trust, Westminster Bridge Road, London SW1 7EH, UK; 2 Cleveland Clinic London, 33 Grosvenor Place, London SW1X 7HY, UK; 3 National Heart & Lung Institute, Hammersmith Hospital, Imperial College London, London SW7 2BX, UK; 4 St Thomas’ Hospital, Guy’s and St Thomas Hospitals NHS Trust, Westminster Bridge Road, London SW1 7EH, UK; 5 Hammersmith Hospital, Imperial College Healthcare NHS Trust, Du Cane Road, London W12 0AH, UK

**Keywords:** atrial septal defects, complex anatomies, 3D transesophageal echocardiography, transcatheter ASD closure

## Abstract

Transcatheter secundum atrial septal defect (ASD) closure is the preferred approach in the majority of cases. Building skill sets through understanding complex ASD anatomies is essential. Such anatomies include ASD associated with aneurysmal septum, multiple defects, absence of anterosuperior or posteroinferior rim and malaligned septum This expert-based review will focus on the key role of advanced TOE imaging during transcatheter ASD closure in challenging anatomies. We describe our institutional experience and provide a practical approach of how to plan and navigate device choice and its delivery to ensure optimal outcomes.

## 1. Introduction

Transcatheter atrial septum defect (ASD) closure is the preferred approach in the majority of cases with secundum type ASD [1]. Avoiding cardiac surgery, sternotomy and cardiopulmonary bypass along with rapid recovery times and reduced in-hospital stay marks transcatheter atrial septum defect closure (tASDc) as the first-line standard treatment. Feasibility and procedure success depend on a number of factors including anatomical suitability (size and number of defects, rim stability and length), device size and characteristics (engineering concept of self-centering versus non-self-centering [2]), associated defects (where surgery may be more appropriate to address all pathologies), physiology (abnormal left or right ventricular compliance or pulmonary hypertension) and potential for complications. Appropriate case selection ensures complication rates of 1% or less [1].

Single secundum ASDs with firm adequately sized rims (≥5 mm) of moderate size or less (≤25 mm, comfortably within device range) are considered straightforward to close. Complex anatomies include ASD associated with aneurysmal septum (hypermobile floppy rims), multiple defects (including discrete or small multiple fenestrations), absence of anterosuperior or posteroinferior rim and malaligned septum (where there is septal tissue separation), for further explanation see Table 1. Device sizing and positioning present unique challenges with higher risk of device embolization, residual leaks and erosion. Our institutional experience with three-dimensional echocardiography (3DE) has significantly improved our ability to successfully treat complex anatomies. Awareness of the potential risk factors for complications improves device selection and decision making at the time of tASDc [3]. This expert-based review will focus on the key role of advanced TOE imaging during tASDc in challenging anatomies. We describe our institutional experience and provide a practical approach to each complex ASD phenotype. We include case illustrations performed by the authors.

## 2. Role of Advanced Transesophageal Echo Imaging Guidance

**Figure 1:**
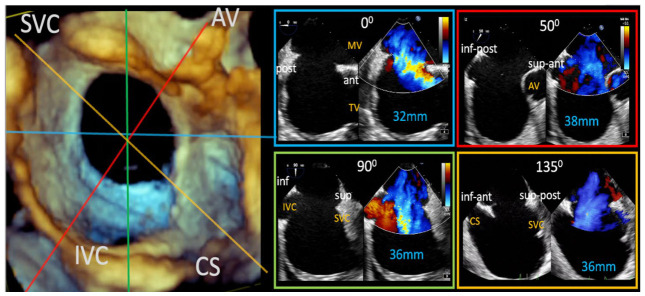
**TOE protocol for ASD assessment.** 3D echo image (3D live zoom mode) en faces right atrial (RA) view with landmarks. Left panel depicts the four standardized views. Each coloured box relates to each coloured line 0, 50, 90, 135 degrees, which demonstrate the key anatomical landmarks in each view. The measurements of the ASD are shown, colour Doppler is applied and the largest diameter of the defect is found (typically around end ventricular systole) in the cardiac cycle and the measurement is made from edge to edge of the colour flow image. The measurements are then averaged. For device sizing see Table 2 and Figure 3. The ASDs depicted have minimal rims for much of its circumference and was deemed unsuitable for transcatheter device closure. *SVC superior vena cava, AV aortic valve, CS coronary sinus, IVC inferior vena cava, MV mitral valve, tricuspid valve*.

**Figure 2:**
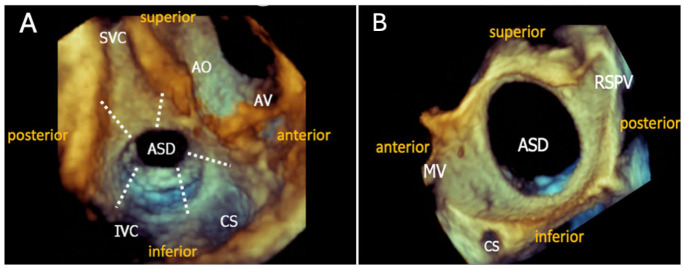
**3D echo views.** Correctly orientated 3D echo en face views and depicts key rims of the ASD and landmarks. Image (**A**) right atrial en face view. The white dotted lines depict the rims, each rim approximately measures 20% of the ASD circumference. Image (**B**) left atrial en face view. *Ao* aorta, *AV* aortic valve, *CS* coronary sinus, *IVC* inferior vena cava, *SVC* superior vena cava, *MV* mitral valve, *RSPV* right superior pulmonary vein, *ASD* atrial septal defect.

Good knowledge of the principles of the procedure, recognition of complex morphologies, planning of device type and size, method of deployment and post-deployment checks are all essential steps [4]. Significant advances in echocardiography imaging and in particular 3DE has improved our understanding of complex morphologies [5,6,7]. 3DE allows an instantaneous appreciation of defect position, orientation, size and spatial relationships to surrounding structures. This comprehensive anatomical understanding provides insights into specific ASD characteristics and the interactions required for optimal device selection. Further, following device deployment, 3DE provides invaluable information superior to two dimensional (2D) imaging. The latest echo software permits seamless 3D multi-plane reconstruction (MPR) in real-time. This simplified method affords precise assessment of device conformity, alignment and correct capture of the rims. Thereby reducing both uncertainty and time required in determining a satisfactory deployment. Where deployment is suboptimal, 3DE can highlight crucial information not apparent with 2D imaging [8]. Therefore, experience in 3D transesophageal echocardiography (TOE) is essential. Table 2 summarizes our approach to optimal 3DE data acquisition. Correct image orientation enables precise dialogue between the interventionist and imager and is therefore encouraged. A systematic approach to assessment of the atrial septum (Figure 1) has previously been discussed elsewhere [9]. The assessment should include correctly orientated 3D en face views of the left and the right atrial septum (Figure 2). While used less frequently, other methods of echocardiographic imaging may be utilized (transthoracic echocardiography and intracardiac echocardiography) and are dependent on available resources, operator experience and patient cohort [10]. This article will focus on TOE, although the general principles described apply to all echocardiographic modalities.

## 3. Secundum ASD Complex Anatomies

### ASD with Absent Rim

**Figure 3:**
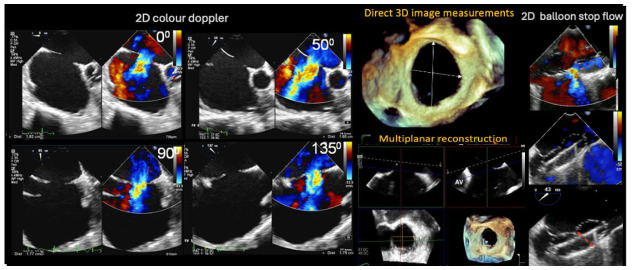
Methods for secundum atrial septal defect (ASD) device sizing. From left to right: 2D colour measured in the four standard planes with colour Doppler; largest ASD dimension in cardiac cycle (around end ventricular systole); measure from edge to edge of the 2D colour flow; the formula used to calculate the device size is given in Table 2. The second method uses 3DE imaging, 3D live zoom mode (see Table 1) acquisition and either a direct measure in still frame where ASD appears largest (long and short axis dimensions) or using multiplanar reconstruction (see Table 2). Lastly using 2D echo colour Doppler placed over the ASD/septum, a balloon is gradually inflated and colour flow is monitored until the flow stops (no longer seen within edges of balloon and defect is fully occluded); this method will ensure septum is not overstretched and ASD device oversized.

Incidences of device erosion are rare, 0.1–0.3% [11,12,13], and may present with chest pain, dyspnea, and hypotension due to hemopericardium, tamponade, aortic fistula and death [12]. Most erosions occur early after device deployment typically within 12 months. Although late erosions have been reported (as late as 8 years). Following initial reports, recommendations were made to mitigate the risks of erosion, highlighting echo predictors [14]. Most erosions occurred with deficient anterosuperior rim (aortic rim) and device oversizing. This rim deficiency being the most common. Therefore, the concept of balloon sizing using the colour Doppler ‘stop-flow’ method evolved [15]. Balloon sizing is not mandatory and will depend on the interventionist’s preference, along with the experience of the imager. 2DE using colour flow or 3DE (either direct from the 3D image or using MPR) measurements may also be utilized [16,17,18,19]. In our practice we do not routinely balloon size, we employ a simplified method either with 2D colour or 3D TOE to optimally device size—see Table 3 and Figure 3 for methods. When defect sizing doubt remains then balloon interrogation is the default (or may be the preferred method in all cases in some institutions), assessing not only defect size but rim behaviour and compliance. However, despite mitigating the risk of oversizing, careful consideration on optimal device type and its final positioning in relation to the atrial roof and aorta are crucial [5]. The TOE protocol should include a gradual sweep through the angles to critically assess the absence of an aortic/superior rim and its extent. 3DE imaging will depict the percentage of rim deficient, and whether this extends superiorly. Where no septum secundum tissue is seen, and the aortic wall exposed (termed ‘bald aorta’ [5]), then direct contact of an ASD device will occur. Contra-indications for complex ASD anatomies are summarized in Table 1.

**Figure 4:**
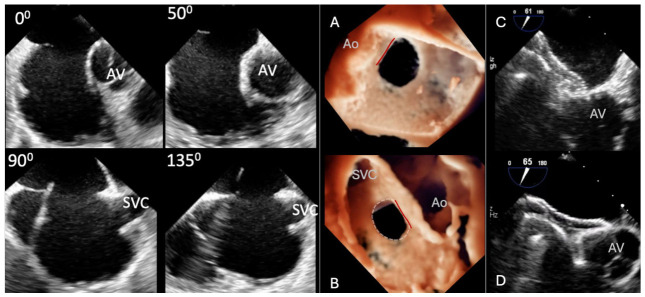
Deficient aortic rim. From left to right, 2D TOE images showing the ASD rims in 0, 50, 90, 135 views. There is no aortic rim. The extent of the absent aortic rim is understood on 3DE imaging en face views (image (**A**) is the LA en face view; image (**B**) is the RA en face view). The curved distance can be traced for the entire circumference of the defect (white dotted line) and the region where the rim is absent (red line) also traced. The latter divided by the circumference will give a percentage, if a significant proportion (in our experience approx. >20%) of the rim is totally absent this may increase the risk of erosion. How the ASD device discs interact with the surrounding tissue and aorta may increase the risk of erosion. Image (**C**) shows both device discs are in contact with the aortic root and during the cardiac cycle appear to push onto the aortic wall. Image (**D**) shows the device discs rest tangentially along the aortic wall, the waist is also in contact with the aortic wall (see text for explanation). SVC—superior vena cava; AV—aortic valve; Ao—aortic root.

Routine procedural imaging steps have previously been described [9]. Specific imaging considerations for deficient aortic rim are highlighted in Figure 4. Assessment includes identification of aortic rim tissue (sufficient ≥ 5 mm, deficient < 5 mm or absent < 1 mm), its extension circumferentially around the aorta (best assessed using en face 3D view from the RA side) and whether the deficient rim extends superiorly to involve the atrial roof. In addition, it is important to ensure there is no malalignment of the septum primum attachments to the secundum septum (see section below). In our practice, device sizing can be performed using the methods described in Table 3 and Figure 3.

**Figure 5:**
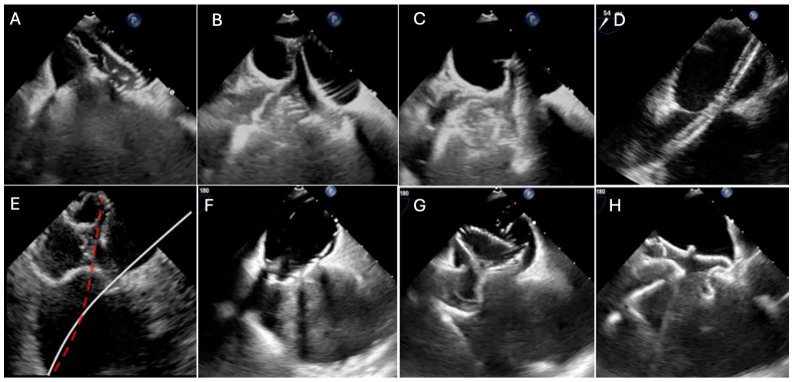
ASD closure using balloon assisted technique. Images (**A**–**C**) demonstrate the large LA disc prolapsing into the RA. The aim is to image the entire length of the sheath and its relationship to the septum, image (**D**). This will aid the interventionist in understanding how to manipulate the sheath and the positioning of the LA disc during deployment. Image (**E**) depicts the sheath position (white line) when the device was deployed and explains why the disc prolapsed into the RA. The sheath is rotated to direct it posteriorly and superiorly (red dotted line) within the LA, so allowing the LA disc to be coaxial during deployment and able to catch the aorta. Despite these maneuvers the device failed to deploy correctly. Images (**F**–**H**) show an inflated balloon across the septum; the device is gradually deployed while the balloon gradually deflated to allow the LA disc to position coaxial with the septum, and the deployment is then completed.

A recognized challenge is seen during deployment where the device may continually prolapse from the LA into the RA, due to lack of sufficient aortic rim and angle of approach. Several additional steps may be attempted. The aim is to optimally align the delivery sheath so it is as coaxial as possible to the septum. This way when the LA disc of the device is exposed and brought back to the septum it catches the aorta without prolapsing into the RA. Such maneuvers include: rotation of the delivery sheath to point superior and posterior to allow better coaxial alignment with the septum; reshaping the sheath; device waist deployment in the LA to encourage LA disc to catch the aorta; right upper pulmonary vein technique to optimize delivery of the LA disc capture of the posterior-superior rim and prevent prolapse into the RA; and balloon assisted deployment where the balloon displaces the LA disc superiorly and posteriorly in the LA allowing better coaxial alignment of the device to the atrial septum [20,21], (Figure 5). During deployment the sheath should be imaged in its entirety (optimized by altering the image plane angle typically somewhere between 0 and 70 degrees) as it travels from the IVC across the RA and atrial septum into the LA. If the sheath position is difficult to locate then biplane imaging can be used to find the optimal view (independently altering the angle rotation of the right image to find the sheath in its entirety). Monitoring the device deployment and the relationship of the sheath and the LA disc to the atrial septum will help determine what steps are needed to enable a coaxial deployment of the LA disc, see Figure 5 and Table 4.

The characteristics of the device appearance in relation to the aorta and atrial roof may help highlight high risk appearances necessitating device repositioning, resizing or perhaps even removal, see Figure 5 and Table 1 for contra-indications. These include the device discs pointing into and partially distorting the aorta, the device discs straddling the aorta but not tangentially aligned to the aorta, and dynamic cardiac motion with a device waist abutting the aorta and swaying in a see-saw motion.

Other relevant rims include deficient superior and inferior-posterior rims. The former may result in direct device contact with the atrial roof. If accompanied by aortic root rim absence, it may be associated with increased risk of erosion. Early case reports noted absent superior rim to be a high-risk feature where the erosion occurred into the atrial roof on the RA side. Careful interrogation of the free walls of the LA and RA both superiorly (roof) as well as in the anterior and posterior regions is essential to ensure there is no disc contact. If the discs straddle the walls and are not directed into the wall (similar appearances to the aorta assessment) then it may be feasible to leave the device. Posterior and inferior rim deficiencies may be less easily imaged. If there is extensive rim deficiency involving most of the rim (>20% of circumference in our experience), tASDc may not be suitable due to significant risk of embolization. Advanced technique involving a combination of a covered stent placed at the orifice of the IVC (or SVC orifice in the case of absent SVC rim) and then deploying an ASD device has been described [22]. The inferior region can be difficult to visualize on TOE due to a small LA and a large RA. Further compounded by the close proximity of the inferior-posterior septum to the TOE probe. This can be overcome with retroflexion of the TOE probe. This increases the distance between the atrium and probe [23]. 3D imaging and MPR methods will give a comprehensive understanding of the extent of rim deficiency and rim length if present.

Once the device is deployed, 2D with colour Doppler and 3D imaging, will allow step-by-step interrogation of the deficient rim and its relationship to the device. 3DE, including MPR, can provide on-axis imaging of device alignment to the surrounding structures and available space from both LA and RA views. This is invaluable in determining whether optimal deployment has been achieved and if any high-risk features exist (see Table 4). Such features include device protrusion into a wall, incomplete capture of septal tissue within the device discs and residual flow seen outside the device. Once the device is deployed, a similar 2D with colour and 3DE imaging protocol is performed to confirm a satisfactory final position.

## 4. ASD with Malaligned Septum

**Figure 6:**
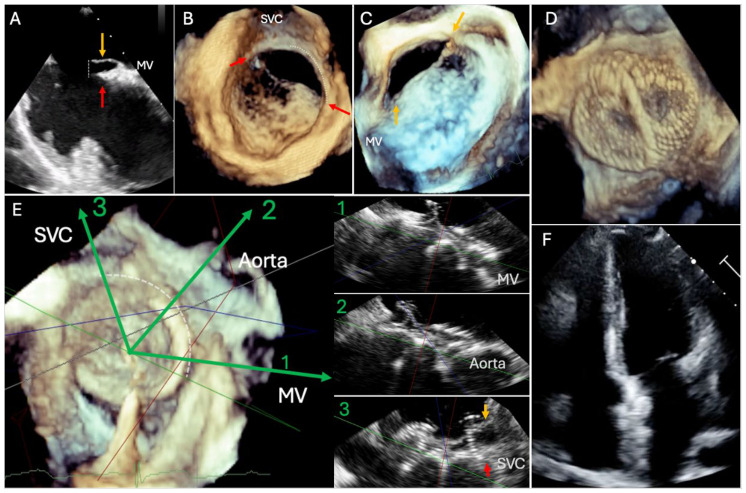
**Malaligned septum.** Image (**A**) depicts 2D 0-degree view of the atrial septum; septum primum (yellow arrow) is separated from the plane of the septum secundum (red arrow). The tissue separation measures 7 mm. In image (**B**), RA en face view, and image (**C**), LA en face view, the white dotted line depicts the off-set septum secundum rim from the RA side (between red arrows), and the septum primum tissue attachment on the LA side (yellow arrows). The defect was balloon sized (stop flow technique) and a device size chosen as balloon waist diameter +4 mm. Image (**D**), device deployed LA view. Image (**E**), careful step-by-step interrogation using multiplanar reconstruction of the 3D image to ensure that both primum and secundum tissue have been captured between the device discs; sweep from MV to SVC 1–3 (image 3 shows primum yellow arrow and secundum, red arrow, with LA and RA device discs positioned correctly). Image (**F**), follow up transthoracic echo at six months depicts good device position and reduction in right heart size. SVC—superior vena cava; MV—mitral valve.

Septal malalignment is a relatively infrequent finding during ASD closure. A precise definition is lacking and a variation in terms in the literature appear to describe similar entities including spiral, off-set and double layer atrial septum [24,25,26]. The unifying anatomical characteristic is malalignment or displacement of the septum primum (SP) towards the left atrial side, which is separated from the septum secundum (SS), (Figure 6). Risks arise when the defect is inappropriately sized due to the off-set nature of primum and secundum tissue. The degree of widest separation must be appreciated to ensure the device is correctly sized. In our experience if the separation is >8 mm then the discs will splay widely due to a broader waist as they negotiate this separation. This will result in a degree of ‘cotton reeling’ of the device. In this setting the device should be intentionally sized up 1 or 2 sizes depending on the degree of septum separation. When deploying the device care must be taken to ensure the LA disc has fully captured the SP tissue and resting fully within the LA. Similarly for the RA disc, this should rest fully in the thickened SS entirely within the RA. Risk of embolization typically occurs when one of the device discs is inadvertently placed within the space between the two layers of tissue, or the device has been wrongly sized. Careful 3DE assessment is essential to evaluate the position of both discs in their entirety using the en face LA and RA views. Further corroboration should be sought with 2D and colour Doppler assessment and then 3D MPR with a step-by-step 360 sweep rotating around the centre of the device viewing in detail both layers of tissue (SP and SS) positioned between the discs, see Figure 6 and Table 4. Some limited flow may be seen on colour Doppler particularly at the anterior rim, but as long as good coverage of the defect has been achieved by both discs and device stability confirmed then the device can be deployed.

## 5. Aneurysmal Septum ASD and Multiple Defects

**Figure 7:**
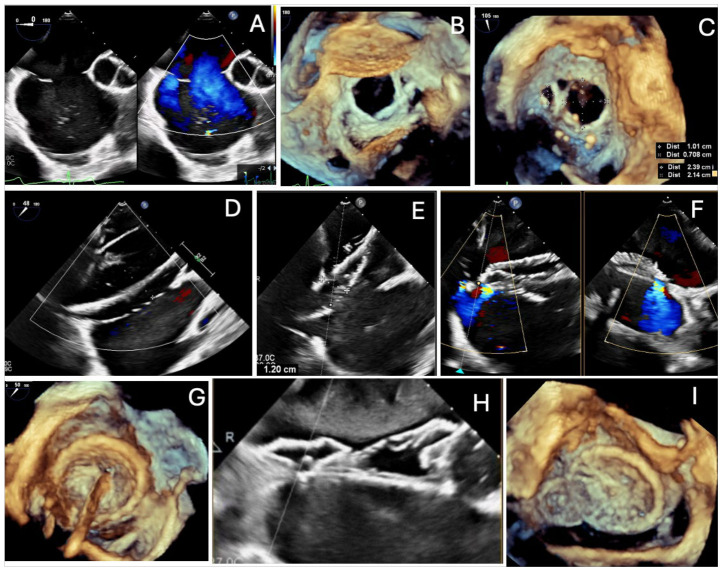
**ASD with multiple defects.** Image (**A**), 2D TOE with colour Doppler showing two defects. Images (**B**,**C**) are en face LA and RA views respectively, with direct 3D measurements of the two largest defects. Images ((**D**,**E**) show two wires placed through each larger defect and balloon sized using stop flow technique (larger defect 30 mm, smaller defect 12 mm). A 33 mm Figula Flex II Occlutech (Occlutech ASD Occluder; Occlutech, Helsingborg, Sweden) device deployed first and not released, images (**F**,**G**). Then 13.5 mm device was deployed with careful adjustments to attempt to interlock the devices. Images (**H**,**I**) show deployed devices after release.

**Figure 8:**
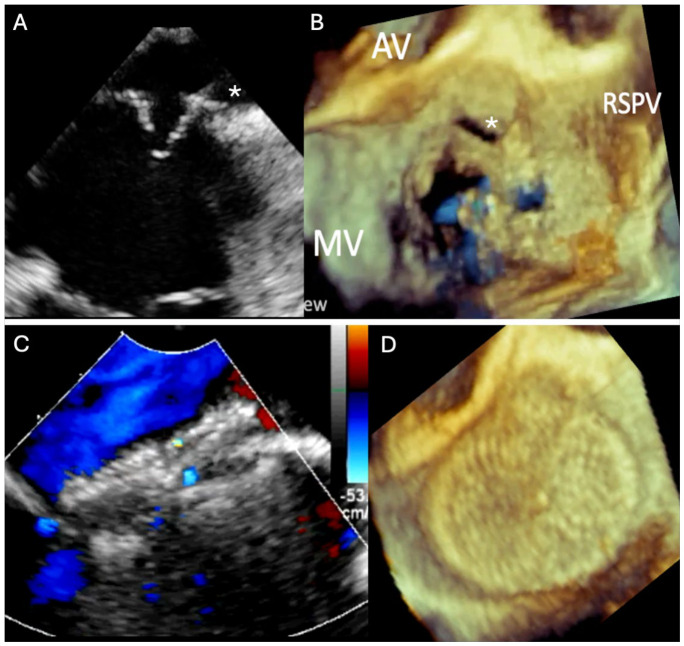
Fenestrated aneurysmal ASD. Image (**A**), 2D TOE, and image (**B**) 3D LA view, depict hypermobile septum with defects within primum septal tissue and also a PFO (white star). The total septum length was measured directly using the 3D images and a non-self centering device was sized for the disc size to cover as much of the septum as possible within the available space. A device of equal disc sizes with a linear waist connection was placed through a central fenestration. Prior to release the degree of mobility and relationship of the discs to all rims, in particular the aorta, were carefully assessed. Images (**C**,**D**) show the device seen on 2DE and 3DE after release. AV—aortic valve; MV—mitral valve; RSPV—right superior pulmonary vein.

Transcatheter ASD closure in the presence of aneurysmal SP may increase the risk of device embolization. 3DE gives important insight into the overall size of the fossa ovalis (FO) and extent of hypermobile SP tissue as well as its attachments to SS, see Figure 7. It also depicts position and number of defects. Image resolution can at times be challenging when SP tissue is thin and mobile. The use of photorealistic imaging (Table 2) may circumvent this limitation. However, to accurately assess all defects 2D and 3D colour imaging is needed. Reducing the colour scale may improve defect identification. However, it may produce colour artefacts and confuse the assessment. Attempting image optimization by adjusting the gain, focus, reducing sector width and harmonic settings can improve image resolution. Use of multiple echo windows and reducing 3D zoom and 3D live sector acquisitions (increasing frame rates) will allow systematic septum interrogation to aid assessment, see Table 4. Where a single defect is found and, despite a hypermobile SP, the tissue attachments of the ASD are fixed without excessive motion, then standard echo measurements may suffice. In the case of hypermobile SP tissue involving the ASD margins, how much the ASD will fold back and open wider is unpredictable [27]. Wire passage through the defect gives important insight into any configuration change. Direct 3D dimension measurements can be repeated to re-assess if the defect appears larger. If doubt remains, then balloon sizing using the colour Doppler stop flow technique is necessary. For such complex defects it is our preference to balloon size. This gives us additional valuable information as to the compliance and behaviour of the defect and surrounding structures. In the case of multiple defects, attempts are made to cross the largest ASD. If multiple fenestrations then we aim for a central defect. The position and which defect has been crossed can be confirmed with 3DE imaging. Wire passage can be viewed from the RA side and followed across into the LA side. In the setting of a fenestrated hypermobile septum attempts should be made to cover the entire FO where possible—see Figure 8. This will ensure all defects identified or unidentified will be covered. In addition, the mobile septal tissue is captured allowing the device discs to rest on the secundum septum, providing stability. In such cases we measure the length of the septum in transverse and longitudinal planes (0 and 90). This can also be directly measured from the en face RA/LA 3DE views when the septum looks largest (ventricular systole). The typical ASD device has a large waist. Its diameter is the given device size. ASD closure concept is based on filling the defect with the waist of the device. Hence it is a self-centering device. In the case of very small defects (<5 mm) a non-self-centering device, such as Occlutech Uni (Occlutech UNI Occluder; Occlutech, Helsingborg, Sweden), Amplatzer Cribriform (Amplatzer Septal Occluder Cribriform; Abbott, St. Paul, MN, USA) or Gore Septal Occluder device (GSO, W.L. Gore & Associates, Newark, DE, USA) may be preferred if the disc sizes will cover the entire FO. For larger defects, individual balloon sizing is performed and, if appropriate (defects >7 mm apart, since the majority of self-centering ASD devices used have a LA disc size typically 7 mm wider than the waist), separate devices are attempted. Where the distance separating defects is <7 mm then balloon sizing with a degree of over stretching is considered. In this instance attempts are made to cover adjacent defects. If residual defects are left, then additional devices may be deployed. A general principle when using non-self-centering devices of any type is the disc size should be at least double the size of the defect crossed. Such devices may rest at one edge of the defect and unless the disc radius is at least 1–2 mm larger than the diameter of the defect there is a risk of a residual leak. The advantage being a larger percentage (if not all) of the FO will be covered.

Prior to release, careful assessment of device mobility and its relationship to the aorta is essential to avoid the risk of complications. Device embolization may occur in this anatomy where one disc is inadvertently partially deployed on the same side as its counterpart. This is a risk associated with the hypermobile nature of the septum. Again, 3DE assessment is necessary to assess disc positioning in relation to the septal tissue, Table 4.

## 6. Very Large Secundum ASD

In the setting of a very large ASD ≥ 30 mm, visualization of the defect in its entirety can be challenging. This is compounded by the relatively small LA size and, therefore, echo window sector width. Careful 2D and 3D systematic visualization of the ASD rims in sections will allow evaluation. Our practice is to use 3D live format to manually widen the sector widths in lateral and/or elevation planes with multi-beat acquisitions (to improve resolution) to visualize and assess step-by-step the entire atrial septum. Limitations in device closure include significant proportion of the deficient rim (continuously absent along 40% or more of the circumference in our experience, which equates to two adjacent rims being involved) and defect size being too large for the available devices [28]. The largest available balloon sizing diameter is 34 mm and, therefore, limits this technique for defect sizing [29]. When used the balloon may not form a clear waist, creating uncertainty as to where to measure the balloon diameter. If an attempt at closure is felt feasible, the widest diameter may be used to device size or an estimation taken from the entire septal length and, therefore, available space to determine device size. However, in most cases surgical closure is the preferred option.

## 7. Follow-Up and Device Surveillance

Post tASDc imaging follow-up is essential when performing complex ASD closure. Potential complications can be assessed with transthoracic echocardiography (TTE). In cases of ‘bald’ aorta with deficient rim >20% (in our experience) or high-risk echo features more intense follow up may be preferred. Assessing device position and correct alignment with the atrial septum, residual abnormal flow (colour Doppler flow around and outside the device) and presence of pericardial effusion may be early warning signs of device complications.

Typical practice includes a TTE immediately post procedure, followed by 4–6 weeks check to ensure device position and absence of other complications. Then a further comprehensive assessment at 6–12 months which should include right heart size, function and pulmonary pressure measurements [3]. In select cases with potential high-risk echo features, additional assessment may be needed at 1 week, 1 month, 6 and 12 months and, if concerns remain, annually thereafter [30]. Ideally prior to device closure, in the presence of complex anatomy, the pros and cons should be discussed with the patient. Post procedure (as would be the case in all patients), careful counselling of concerning symptoms (chest pain, syncope, fevers) and importance of early or urgent review should be emphasized.

## 8. Conclusions

Transcatheter closure is the preferred approach in the majority of secundum atrial septal defects. Advanced echocardiography, and in particular 3D imaging, is pivotal in understanding ASD morphology. Complex anatomies encountered include deficient rim, malaligned septum, multiple defects and aneurysmal atrial septum. 3D echo formats (biplane imaging, live 3DE and MPR mode) provide a comprehensive assessment of the entire defect, its surrounding anatomy and allow precise planning of device type, device size and method of deployment. Careful assessment using 3DE of the device prior to and post-release can be performed with confidence and may reduce the risk of device embolization, residual leaks and erosion. As transcatheter therapies continue to evolve and complex anatomies increasingly treated, advanced echo techniques are proving to be an essential tool to their success.

## Figures and Tables

**Table 1:** Simple versus complex secundum ASD anatomies. Algorithm to aid decision making for suitability for device closure.

**Simple Secundum ASD**
Predictable device deployment: Single defect. Measures ≤ 25 mm. * Sufficient rims, ≥5 mm. * Moderate complexity 26–29 mm due to device size and potential need for modified procedural techniques
**Complex secundum ASD**
Potentially device-closable: Expert centre decision. Higher complication risk awareness. Need for modified procedural and advanced imaging techniques. Large defect ≥ 30 mm. Hypermobile septum, septal excursion in relation to either side of the midline ≥ 10 mm. Malaligned septum, septal separation of septum primum and secundum tissue. Deficient rim, aortic rim deficiency most commonly encountered. Multiple defects, discrete or multiple small fenestrations.
**Relative contra-indications to device closure**
Multiple defects with limited stable rims, risk the device may dislodge. Malaligned septum with septal separation ≥12 mm **^§^**, risk the device may embolize. Absent aortic rim, device waist and discs in direct contact with aorta and dynamic motion, risk of erosion. Deficient infero-posterior rim poorly visualized due to limited echo windows, risk the device may embolize. Complete absence of IVC or SVC rim, expert centre referral for stent + ASD device may be possible.
**Absolute contra-indications to device closure**
Very large ASD > 38 mm, too large for available device. Total absence of 2 rims (≥40% of ASD circumference) **^§^**. Malaligned septum with significant septal separation > 15 mm **^§^**. Absent aortic rim where device disc protrudes into/distort aorta, despite attempts to optimize device alignment.

^§^ numeric threshold given are guide values and these reflect the authors’ experience.

**Table 2:** 3DE image acquisition of the atrial septum.

**ECG and Echo Window**
ECG leads attached, with stable ECG recording. Ensure left atrium is optimally volume filled. Consider giving IV fluids providing that LV/RV function are preserved. Optimize echo window, typically mid-oesophageal best, if limited window then high or low oesophageal windows may be helpful.
**TOE 50-degree view**
For ease, acquisition at 50 degrees ensures a standardized method for 3D image manipulation, as well as maintaining familiar imaging planes in MPR mode.
**3D/4D Zoom mode**
Go to Zoom mode, left image (lateral plane) is the 50 view, orthogonal right image (elevation plane) is the 135 view in the correct orientation (SVC to right of screen). Widen the area of interest box to include entire atrial septum and a little of the LA and RA, in both lateral and elevation planes, throughout the entire cardiac cycles. Ensure landmarks are included (Figure 2) SVC, IVC, CS, aorta, MV, as this will help orientate the image.
**Image orientation**
Once sectors optimized, activate live zoom mode. Using the trackball pull down the 3D image. The LA en face view of the atrial septum will be visible. Anti-clockwise rotate the image (Z rotate) to position the MV at 8 o’clock.
**Image optimization**
Ensure focus, gain (and harmonics, if desired) settings are optimized. Acquire the image (ideally 2 cardiac cycle acquisition). Now flip the image 180 degrees right to left. The RV en face view is now visible. Minor adjustments with Z rotate may be needed to bring the SVC orifice to 11 o’clock (Figure 2). Acquire the image. 3D colour Doppler imaging can also be obtained with the same datasets.
**Data analysis**
En face views of the LA and the RA can now be analyzed. ASD border detection can be enhanced with photorealistic light source and Glass view formats. The 3D images can be analyzed using multiplane reconstruction, and a step-by-step segmental analysis of the ASD, size, rims and other relevant features can be assessed.
**Additional considerations**
Usually due to limited movement of the septum, single beat 3D acquisition is adequate for detailed image resolution (providing > 8 Hz). If not adequate, then reducing sector width or multi-beat acquisition may improve image resolution. Breath-holding may be needed in the latter. The primum septum is often very thin tissue and 3D echo drop-out is common and should not be confused with additional defects. 2D and 3D are complimentary tools and if in doubt assess the region concerned in the appropriate 2D TOE views and with colour Doppler (reducing the colour scale can be helpful from 50 to 60 down to 30–40 cm/s).

*IV* intravenous, *LV* left ventricle, *RV* right ventricle, *MPR* multiplane reconstruction, *LA* left atrium, *RA* right atrium, *Ao* aorta, *AV* aortic valve, *CS* coronary sinus, *IVC* inferior vena cava, *SVC* superior vena cava, *MV* mitral valve, *RSPV* right superior pulmonary vein, *ASD* atrial septal defect.

**Table 3:** 2D and 3D TOE device sizing methods, see also Figure 3.

**2D ECHO METHODS**
**2D colour Doppler method** Use standard colour scale. Scroll through cardiac cycle, locate largest dimension in cycle. Measure inner edge-inner edge of colour Doppler flow map at level of ASD If all dimensions within 2 mm, use largest dimension = 2D colour dimension. Or if >2 mm then average all four dimensions = 2D colour dimension. 2D colour dimension +20% = device size. No aortic rim, 2D colour dimension +25% = device size. Aneurysmal septum, 2D colour dimension +25% = device size.
**2D echo balloon sizing method** Stop flow technique. Optimize 2D image to align with shaft of balloon typically 50 degrees TOE view. Place colour flow Doppler across entire ASD and monitor slow balloon inflation. Stop balloon inflation when colour flow through ASD no longer seen. Measure balloon waist on 2D once flow stops. Balloon waist dimension = device size. If no aortic rim, balloon waist dimension +2–4 mm = device size. Aneurysmal septum, balloon waist dimension +2–4 mm = device size.
**3D ECHO METHODS**
**General principles** Scroll through cardiac cycle to locate largest dimension in cycle. Measure inner edge to inner edge, long and short axis. Average the two dimensions. Averaged dimension = device size. If no aortic rim, average dimension + 4 = device size.
**Direct measurements from live 3D image method** Long axis and short axis dimension. Average measurements.
**3D multiplanar reconstruction method** Freeze image and scroll to find largest ASD frame use live 3D image. Align blue line along plane of septum in red and green boxes. Place red and green lines within blue box in centre of ASD and rotate planes to optimize for long and short axis of ASD, measure dimensions.

*ASD* atrial septal defect, *TOE* transoesophageal echocardiography.

**Table 4:** Imaging assessment, high risk echo features and optimal device positioning.

**Imaging Tips and Tricks for Transcatheter Device Closure of Complex ASD**
**DEFICIENT RIM**
**Imaging assessment** *Aortic rim:* <5 mm SP tissue seen adjacent to aorta. TOE 50 degree (SAX-AV) view. Sweep through angles from 30 to 90 to assess entire aortic rim border. 3DE zoom mode with MPR provides overview of entire rim. Use standard defect sizing methods (Table 2). *Superior Rim:* <5 mm SP tissue adjacent to SVC. Standard sizing methods. *Infero-posterior Rim:* <5 mm SP tissue adjacent to IVC. Challenging to image, mid to deep oesophageal view with retroflexion of the probe +/− leftward tilt to optimize for IVC orifice. 3D imaging ideally required to allow detailed assessment of entire inferior atrial septum between IVC and CS. Similar assessment for posterior septum between IVC and SVC. Standard sizing methods, unless doubt remains on true extent of inferior-posterior rim, where oversizing device may be required, as higher risk of device embolization has been encountered.
**High risk echo features and device sizing considerations** Complete absence of SP (septum primum) tissue when aortic wall is exposed to atrial cavity (termed ‘bald aorta’). Deficient aortic rim >20% of ASD circumference * (increases risk of device waist contact to exposed aortic wall and erosion). Caution with absent inferior rim as high risk of embolization.
**Optimal device positioning** *Aortic rim:* Ensure: - Neither discs point directly towards aorta (particular risk is the RA disc). - If straddling of both discs has occurred to anchor device, then ensure discs are directed tangentially to axis of aorta. - Avoid leaving a device if hyperdynamic cardiac motion with device waist abutting aorta and/or discs (described as a see-saw motion of device against aorta) as there is potential for erosion. *Superior Rim:* Careful interrogation of the SVC rim starting from the SVC at 90–110 and gradually rolling the probe towards the aorta as well as gradual incremental reduction in the degrees towards the AV-SAX view (approx. 50 degrees). 3DE and MPR allows a comprehensive assessment. *Infero-posterior Rim:* Challenging to image inferior-posterior rims, mid to deep oesophageal views needed in vertical plane (90 degrees) with sweep of probe right to left to visualize entire rim. 3DE imaging both zoom mode and live imaging will help further elucidate rims.
**SEPTUM MALALIGNMENT**
**Imaging assessment** Separation between SP and SS tissue (SP is displaced into LA). Typically seen superior ASD border and may extend anterior and posterior. Careful interrogation in all views, and extent of separation can be described by assessment 0, (4C), 50 (AV), 90 (bicaval),135 and how many rims are involved. Separation > 8 mm *, may need to upsize device, as potential risk of embolization. Best viewed with 3DE to understand position and extend of SP/SS separation.
**High risk echo features and device sizing considerations** Significant separation > 12–15 mm * of SP and SS tissue, device closure with appropriately sized device may not be possible as device may cotton reel and embolize. Significant oversizing can result in risk of erosion (see deficient rim section). Device sizing should be for SP measurements. Balloon sizing with stop flow technique may be preferred in this anatomy.
**Optimal device positioning** Ensure entire SP has been captured on LA side. Ensure RA disc fully covers RA side and the SP/SS tissue is sandwiched between the two device discs. 3D MPR will allow a comprehensive assessment. Assess superior-anterior rims carefully as well an any region of deficient rim to ensure device discs do not impinge into atrial wall or aorta inappropriately (see deficient rim section).
**HYPERMOBILE SEPTUM PRIMUM** (aneurysmal septum)
**Imaging assessment** Hypermobile SP tissue that constitutes the fossa ovalis (FO). Use colour compare setting to view 2D anatomy with any flow through the septum. If unsure then cautious reduction in colour scale to elucidate turbulent flow through the septum can be performed (colour scale 30–40 cm/s). 3DE can give a comprehensive overview of size of FO and extent of hypermobile septum tissue and how this relates to any defects seen (3DE colour may be useful and can be optimized with multi-beat acquisition). Measure entire FO (0 and 90 views) or more accurately on 3DE imaging.
**High risk echo features and device sizing considerations** Single large defect with hypermobile septum, ballon sizing may be necessary if margins of ASD are flimsy and very mobile. Fenestrations (often multiple, inferior-posterior positioned fenestrations can be challenging to locate) within FO. Strategy may be either to place single non-self-centering device as centrally as possible with the aim of the equal sized discs to cover entire FO or if FO is too large then positioning 2 small discs interlocked to cover entire septum. If neither strategy is suitable, then in some cases if a larger defect is seen (not fenestrations) stretched balloon sizing may allow an ASD device to be placed to cover the majority of the septum.
**Optimal device positioning** Ensure device discs have captured septum secundum ensuring device stability with limited motion. If excessive device motion: - Embolization may be a risk in the case of an ASD device under-sized to the defect (hence balloon sizing advised). - Significant device motion may theoretically result in arrythmias or an awareness by the patient. Large device relative to size of entire septum may result in device being wedged and careful evaluation to ensure device discs in all rims are not impinging onto structures including aorta is essential, as potential risk of erosion.
**MULTIPLE DEFECTS**
**Imaging assessment** Assess location number and size of defects, and relationship to rims. Assess distance between defects: - If spaced > 7 mm apart may be suitable for multiple device closure strategy (in the case of self-centering devices, length of available disc from device waist to edge of disc, e.g., Amplatzer ASO and Occlutech Figulla Flex II). - Sizing as per standard protocols, unless flimsy borders of ASD, where BS with SF technique preferred. - If defects spaced < 7 mm apart then may size for largest defect and decide, if feasible, to cover entire septum.
**High risk echo features and device sizing considerations** Hypermobile FO will require balloon sizing.
**Optimal device positioning** Careful interrogation of remaining uncovered FO if present (typically inferior-posterior regions, use appropriate TOE views including 3DE to assess, colour Doppler): - Ensure no additional defects present. - If so management may be dependent on indication for closure (embolic event versus volume overload) and size of remaining defects to determine if further device is necessary. Assess inter-device positioning, if interlocked ensure good alignment of devices with each other, devices not impinging or interfering with surrounding structures, in particular the relationship to the aorta and any other rims/structures in close proximity to devices placed.

NOTE: * numeric threshold given are guide values and these reflect the authors’ practice. *SP* septum primum; *SS* septum secundum; *SVC* superior vena cava; *IVC* inferior vena cava; *CS* coronary sinus; *FO* fossa ovalis; *BS* balloon sizing; *SF* stop flow technique; *4C* four chamber view; *AV* aortic valve short axis view; *MPR* multiplane reconstruction.

## Data Availability

No new data were created or analyzed in this study. Data sharing is not applicable to this article.

## References

[1] English K.M., Espuny-Pujol F., Franklin R.C., Crowe S., Pagel C. (2025). Secundum Atrial Septal Defect Closure in Adults in the UK. Eur. Heart J.-Qual. Care Clin. Outcomes.

[2] Sohn Y.S., Woo J.S. (2026). Contemporary ASD Occluders: Comparative Insights on Each Device. J. Cardiovasc. Interv..

[3] Shaban Q., Hijazi Z.M. (2025). Secundum Atrial Septal Defects in Adults: All You Need to Know with an Emphasis on Outcome. Expert Rev. Cardiovasc. Ther..

[4] Jalal Z., Hascoet S., Baruteau A.-E., Iriart X., Kreitmann B., Boudjemline Y., Thambo J.-B. (2016). Long-Term Complications After Transcatheter Atrial Septal Defect Closure: A Review of the Medical Literature. Can. J. Cardiol..

[5] Silvestry F.E., Cohen M.S., Armsby L.B., Burkule N.J., Fleishman C.E., Hijazi Z.M., Lang R.M., Rome J.J., Wang Y. (2015). Guidelines for the Echocardiographic Assessment of Atrial Septal Defect and Patent Foramen Ovale: From the American Society of Echocardiography and Society for Cardiac Angiography and Interventions. J. Am. Soc. Echocardiogr..

[6] Budts W., Miller O., Babu-Narayan S.V., Li W., Valsangiacomo Buechel E., Frigiola A., Van Den Bosch A., Bonello B., Mertens L., Hussain T. (2021). Imaging the Adult with Simple Shunt Lesions: Position Paper from the EACVI and the ESC WG on ACHD. Endorsed by AEPC (Association for European Paediatric and Congenital Cardiology). Eur. Heart J.-Cardiovasc. Imaging.

[7] Simpson J., Lopez L., Acar P., Friedberg M., Khoo N., Ko H., Marek J., Marx G., McGhie J., Meijboom F. (2016). Three-Dimensional Echocardiography in Congenital Heart Disease: An Expert Consensus Document from the European Association of Cardiovascular Imaging and the American Society of Echocardiography. Eur. Heart J. Cardiovasc. Imaging.

[8] Deng B., Chen K., Huang T., Wei Y., Liu Y., Yang L., Qiu Q., Zheng S., Lv H., Wang P. (2021). Assessment of Atrial Septal Defect Using 2D or Real-Time 3D Transesophageal Echocardiography and Outcomes Following Transcatheter Closure. Ann. Transl. Med..

[9] Rana B.S. (2018). Echocardiography Guidance of Atrial Septal Defect Closure. J. Thorac. Dis..

[10] Gritti M.N., Carere O., McCrindle B.W., Chaturvedi R.R., Benson L.N. (2026). Complications after Percutaneous Device Closure of Atrial Septal Defects in Children: Prevalence, Outcomes and Associated Factors. Int. J. Cardiol. Congenit. Heart Dis..

[11] DiBardino D.J., McElhinney D.B., Kaza A.K., Mayer J.E. (2009). Analysis of the US Food and Drug Administration Manufacturer and User Facility Device Experience Database for Adverse Events Involving Amplatzer Septal Occluder Devices and Comparison with the Society of Thoracic Surgery Congenital Cardiac Surgery Database. J. Thorac. Cardiovasc. Surg..

[12] Divekar A., Gaamangwe T., Shaikh N., Raabe M., Ducas J. (2005). Cardiac Perforation after Device Closure of Atrial Septal Defects with the Amplatzer Septal Occluder. J. Am. Coll. Cardiol..

[13] Amin Z., Hijazi Z.M., Bass J.L., Cheatham J.P., Hellenbrand W.E., Kleinman C.S. (2004). Erosion of Amplatzer Septal Occluder Device after Closure of Secundum Atrial Septal Defects: Review of Registry of Complications and Recommendations to Minimize Future Risk. Catheter. Cardiovasc. Interv..

[14] Amin Z. (2014). Echocardiographic Predictors of Cardiac Erosion after Amplatzer Septal Occluder Placement. Catheter. Cardiovasc. Interv..

[15] Carlson K.M., Justino H., O’Brien R.E., Dimas V.V., Leonard G.T., Pignatelli R.H., Mullins C.E., Smith E.O., Grifka R.G. (2005). Transcatheter Atrial Septal Defect Closure: Modified Balloon Sizing Technique to Avoid Overstretching the Defect and Oversizing the Amplatzer Septal Occluder. Catheter. Cardiovasc. Interv..

[16] Hascoet S., Hadeed K., Marchal P., Dulac Y., Alacoque X., Heitz F., Acar P. (2015). The Relation between Atrial Septal Defect Shape, Diameter, and Area Using Three-Dimensional Transoesophageal Echocardiography and Balloon Sizing during Percutaneous Closure in Children. Eur. Heart J.-Cardiovasc. Imaging.

[17] Gupta S., Sivasankaran S., Bijulal S., Tharakan J., Harikrishnan S., Ajit K. (2011). Trans-Catheter Closure of Atrial Septal Defect: Balloon Sizing or No Balloon Sizing*—*Single Centre Experience. Ann. Pediatr. Card..

[18] Mani A., Harikrishnan S., Sasidharan B., Ganapathi S., Valaparambil A.K. (2023). Utility of 3D Echocardiography for Device Sizing During Transcatheter ASD Closure: A Comparative Study. J. Cardiovasc. Imaging.

[19] Tzifa A., Gordon J., Tibby S.M., Rosenthal E., Qureshi S.A. (2011). Transcatheter Atrial Septal Defect Closure Guided by Colour Flow Doppler. Int. J. Cardiol..

[20] Jung S.Y., Choi J.Y. (2018). Transcatheter Closure of Atrial Septal Defect: Principles and Available Devices. J. Thorac. Dis..

[21] Kammache I., Mancini J., Ovaert C., Habib G., Fraisse A. (2011). Feasibility of Transcatheter Closure in Unselected Patients with Secundum Atrial Septal Defect, Using Amplatzer Devices and a Modified Sizing Balloon Technique. Catheter. Cardiovasc. Interv..

[22] Abdulwahab H., Husain M.R., Khalid K.A. (2022). Feasibility of Transcatheter Closure of Large Secundum Atrial Septal Defect with Absent Superior or Inferior Rim. J. Interv. Cardiol..

[23] Remadevi K.S., Francis E., Kumar R.K. (2009). Catheter Closure of Atrial Septal Defects with Deficient Inferior Vena Cava Rim under Transesophageal Echo Guidance. Catheter. Cardiovasc. Interv..

[24] Vettukattil J.J., Ahmed Z., Salmon A.P., Mohun T., Anderson R.H. (2013). Defects in the Oval Fossa: Morphologic Variations and Impact on Transcatheter Closure. J. Am. Soc. Echocardiogr..

[25] Roberson D.A., Javois A.J., Cui W., Madronero L.F., Cuneo B.F., Muangmingsuk S. (2006). Double Atrial Septum with Persistent Interatrial Space: Echocardiographic Features of a Rare Atrial Septal Malformation. J. Am. Soc. Echocardiogr..

[26] Takaya Y., Akagi T., Nakagawa K., Nakayama R., Miki T., Watanabe N., Toh N., Ito H. (2020). Clinical Significance of Septal Malalignment for Transcatheter Closure of Atrial Septal Defect. J. Interv. Cardiol..

[27] Ewert P. (2000). Morphology of Perforated Atrial Septal Aneurysm Suitable for Closure by Transcatheter Device Placement. Heart.

[28] Backer J.D., Babu-Narayan S.V., Budts W., Chessa M., Diller G.-P., Iung B., Kluin J., Lang I.M., Meijboom F., Mulder B.J.M. (2025). The Task Force for the Management of Adult Congenital Heart Disease of the European Society of Cardiology (ESC). Eur. Heart J..

[29] Faccini A., Butera G. (2018). Atrial Septal Defect (ASD) Device Trans-Catheter Closure: Limitations. J. Thorac. Dis..

[30] Warnes C.A., Williams R.G., Bashore T.M., Child J.S., Connolly H.M., Dearani J.A., Del Nido P., Fasules J.W., Graham T.P., Hijazi Z.M. (2008). ACC/AHA 2008 Guidelines for the Management of Adults With Congenital Heart Disease: A Report of the American College of Cardiology/American Heart Association Task Force on Practice Guidelines (Writing Committee to Develop Guidelines on the Management of Adults With Congenital Heart Disease): Developed in Collaboration With the American Society of Echocardiography, Heart Rhythm Society, International Society for Adult Congenital Heart Disease, Society for Cardiovascular Angiography and Interventions, and Society of Thoracic Surgeons. Circulation.

